# Electronic‐State Modulation of Metallic Co‐Assisted Co_7_Fe_3_ Alloy Heterostructure for Highly Efficient and Stable Overall Water Splitting

**DOI:** 10.1002/advs.202301961

**Published:** 2023-05-23

**Authors:** Xinyu Wang, Xiaoqin Xu, Yao Nie, Ruihong Wang, Jinlong Zou

**Affiliations:** ^1^ Key Laboratory of Functional Inorganic Material Chemistry Ministry of Education of the People's Republic of China School of Chemistry and Materials Science Heilongjiang University Harbin 150080 China

**Keywords:** Co_7_Fe_3_/Co heterostructures, electrocatalytic water splitting, electron delocalization, electron‐rich interfaces, honeycomb‐like graphitic carbon

## Abstract

Manipulating electronic structure of alloy‐based electrocatalysts can eagerly regulate its catalytic efficiency and corrosion resistance for water splitting and fundamentally understand the catalytic mechanisms for oxygen/hydrogen evolution reactions (OER/HER). Herein, the metallic Co‐assisted Co_7_Fe_3_ alloy heterojunction (Co_7_Fe_3_/Co) embeds in a 3D honeycomb‐like graphitic carbon is purposely constructed as a bifunctional catalyst for overall water splitting. As‐marked Co_7_Fe_3_/Co‐600 displays the excellent catalytic activities in alkaline media with low overpotentials of 200 mV for OER and 68 mV for HER at 10 mA cm^−2^. Theoretical calculations reveal the electronic redistribution after coupling Co with Co_7_Fe_3_, which likely forms the electron‐rich state over interfaces and the electron‐delocalized state at Co_7_Fe_3_ alloy. This process changes the *d*‐band center position of Co_7_Fe_3_/Co and optimizes the affinity of catalyst surface to intermediates, thus promoting the intrinsic OER/HER activities. For overall water splitting, the electrolyzer only requires a cell voltage of 1.50 V to achieve 10 mA cm^−2^ and dramatically retains 99.1% of original activity after 100 h of continuous operation. This work proposes an insight into modulation of electronic state in alloy/metal heterojunctions and explores a new path to construct more competitive electrocatalysts for overall water splitting.

## Graphical abstract

Co_7_Fe_3_/Co heterojunction embeds in 3D honeycomb‐like graphitic carbon is constructed as a bifunctional catalyst. What is distinctive in the structure is that metallic Co is devoted entirely to assist the formation of electron‐rich state over interface and delocalize the surface electronic state at Co_7_Fe_3_. This process changes *d*‐band center position of Co_7_Fe_3_/Co and optimizes affinity of catalyst surface to intermediates.

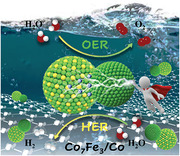

## 1 Introduction

Electrochemical water splitting technology for producing “green hydrogen” is a promising and appealing solution for the global energy scarcity and carbon neutrality.^[^ ^1^ ^]^ Oxygen evolution reaction (OER) and hydrogen evolution reaction (HER) are two half reactions for water splitting and the key factors to obtain clean and high‐purity hydrogen energy.^[^ ^2^ ^]^ Electrocatalysts with decent performance play a pivotal role in the large‐scale implementation of this technology, which are expected to minimize the overpotential necessary to drive the reactions and attain the high current density.^[^ ^3^ ^]^ To date, the state‐of‐the‐art electrocatalysts for OER and HER are Ru/Ir and Pt‐based precious metals, respectively, while their inefficient bifunctional activity, high‐cost, scarcity, and inferior stability restrict their industrial application.^[^ ^4^ ^]^ Therefore, exploring non‐precious and highly efficient bifunctional electrocatalysts for water splitting is still highly desired, which is also envisioned as a necessary part for the future hydrogen economy.

The construction of transition–metal alloys is an efficient strategy to simultaneously drive OER and HER.^[^ ^5^ ^]^ Besides of better electrical conductivity and stability,^[^ ^6^ ^]^ the main principle of alloying is to regulate the electronic states of surface/near‐surface atoms, such as the *d*‐band center position and charge density, which can optimize the adsorption free energy of intermediates on the catalyst surface.^[^ ^7^ ^]^ Thus, alloying catalyst usually presents a better activity compared with those of individual metal elements due to the increased intrinsic activity on each active site.^[^ ^8^ ^]^ More recently, some encouraging studies witnessed this intrinsic activity can be further enhanced by constructing heterointerfaces between an alloy and a single metal.^[^ ^9^ ^]^ For instance, Niu et al. reported the heterostructured Co/CoFe, which can enrich the catalytic active sites and promote the charge transfer between different components, thus improving the electrocatalytic activity.^[^ ^9a^ ^]^ Song et al. developed an interface engineering strategy to fabricate FeCo alloy and metallic Co, which displayed more superior activity and stability to those of commercial catalysts.^[^ ^9b^ ^]^ The interfacial electronic effect was considered to be proficient in adjusting local electronic environments, and promoting the inherent catalytic activity of heterostructures. Still, up to date, the relevant researches are rare and more fascinating insights into the contributions of alloys and single metals on OER/HER, as well as the fundamental mechanism occurring on the interfaces are still ambiguous.

Herein, the integrated Co_7_Fe_3_ alloy and metallic Co heterojunction (Co_7_Fe_3_/Co) embedding in a 3D honeycomb‐like graphitic carbon is purposely constructed as coupled OER and HER catalyst for efficient overall water splitting. The intrinsic correlation between catalytic activity and structural/electronic features is studied based on the experiments and theoretical calculations. For reducing the particle size and exposing more active sites, the honeycomb‐like graphitic carbon with open and well‐developed 3D channels is adopted as a substrate, which not only plays the key role in dispersing and stabilizing the nanoparticles, but also contributes to the nucleation of metallic species. As a consequence, the optimized Co_7_Fe_3_/Co‐600 catalyst with the ultrafine particle size (≈5 nm) and uniform dispersion has exhibited an extraordinary bifunctional activity in alkaline electrolyte with an OER overpotential of 200 mV and a HER overpotential of 68 mV at 10 mA cm^−2^. It is substantially superior to the pure‐phase Co_7_Fe_3_ alloy, and even outperforms most of the reported bimetallic alloy electrocatalysts. Density functional theory (DFT) calculations disclose the integrated Co_7_Fe_3_/Co at the interface site owns the optimized adsorption energies for O‐intermediates and hydrogen, which plays the role as the main active center for OER/HER. Also, the single metallic Co is found to make little contribution to neither OER nor HER. However, after coupling metallic Co with Co_7_Fe_3_ alloy, more electrons on Co layer are deprived and transferred to nearby interface and long‐range Co_7_Fe_3_ layer that not only causes an electron‐rich state at interface, but also promotes the electronic delocalization at the Co and Fe sites in Co_7_Fe_3_ alloy. This process has effectively adjusted the *d*‐center position of Co_7_Fe_3_/Co, enabling it much closer to Fermi energy, and thus strengthening the affinity toward adsorbed intermediates. Besides of the improvement in intrinsic activity, the 3D honeycomb‐like structure with large surface area, high conductivity and good hydrophilicity synergistically acts on the electrocatalytic activity and stability. Notably, Co_7_Fe_3_/Co‐assembled water electrolyzer requires only a cell voltage of 1.50 V to obtain a current density of 10 mA cm^−2^ and maintains 99.1% of initial activity after 100 h of continuous operation. This study highlights the interface effects between an alloy and a single metal, which will provide a new idea to design more advanced bifunctional electrocatalysts for energy conversion devices.

## 2 Results and Discussion

### 2.1 Structural and Morphological Characterizations

**Scheme 1:**
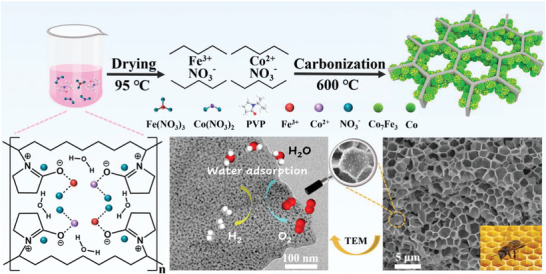
The illustration of design and synthesis of Co_7_Fe_3_/Co by polymer thermal treatment.

**Scheme** **1** shows the synthesis route of heterostructured Co_7_Fe_3_/Co nanoparticles embedding in the 3D honeycomb architecture. Initially, cobalt (II) and iron (III) are bonded through the electrostatic stabilization and coordination between metallic ions and the amide groups on the pyrrolidine rings. Following, the PVP stabilizer is suffered from dehydration (more details see the Thermogravimetric and Differential Scanning Calorimetry (TG‐DSC) curves in Figure S1, Supporting Information) and produces shorter polymer chains, which are capped when they are adsorbed on the surfaces of metallic ions.^[^ ^10^ ^]^ Meanwhile, metallic ions are well dispersed in the networks. The formation of interconnected honeycomb architecture should be attributed to the high viscosity of molten PVP and the gas release from the decomposition of nitrates (fine bubbles) during the following carbonization.^[^ ^11^ ^]^ Figure S2 (Supporting Information) shows the low‐magnification SEM images of Co_7_Fe_3_/Co catalyst, in which the resulted 3D honeycomb structure with regular channels and networks is well displayed at the micron scale.

**Figure 1:**
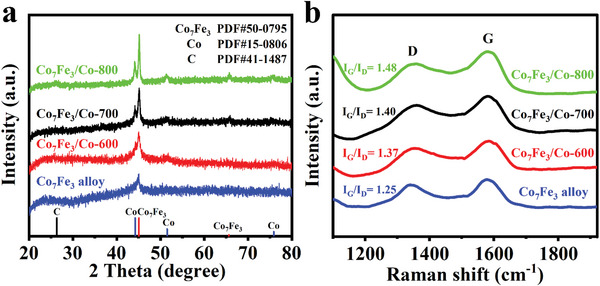
a) XRD patterns and b) Raman spectra of pure‐phase Co_7_Fe_3_ alloy, Co_7_Fe_3_/Co‐600, Co_7_Fe_3_/Co‐700, and Co_7_Fe_3_/Co‐800.

X‐ray diffraction (XRD) is performed to investigate the crystal phase of products derived from PVP‐metal ion precursor. As depicted in **Figure** **1**a, the sample carbonized at 500°C shows the diffraction peaks ≈45.17° and 65.71°, ascribing to (110) and (200) facets of Co_7_Fe_3_ alloy, respectively. As the temperature further increases to 600°C, a series of diffraction peaks for Co are observed at 2*θ* = 44.22° (111), 51.52° (200), and 75.85° (220), revealing the co‐existence of both metallic Co and Co_7_Fe_3_ alloy. The intensity of diffraction peaks is enhanced with the increase of carbonization temperature, implying the increased grain size and crystallinity. In addition, for all the samples, no characteristic peaks for metallic Fe are found, suggesting that the Fe species have been thoroughly alloyed with Co.^[^ ^12^ ^]^ In addition, the XRD peak for graphitic carbon is not apparent until the temperature reaches 800°C, where a weak broad hump of graphite (002) plane appears at ≈26.2°.^[^ ^13^ ^]^ In Raman spectra (Figure 1b), two distinct peaks at ≈1350 and ≈1580 cm^−1^ are attributed to the *D*‐band and the *G*‐band of carbon material, respectively. *D*‐band is caused by the lattice defect of carbon, while *G*‐band is resulted from the internal vibration of the *sp* ^2^ hybridization, reflecting the graphitization of carbon.^[^ ^14^ ^]^ Therefore, the relative intensity of *G*‐band and *D*‐band (*I*_G_/*I*_D_) is commonly adopted to evaluate the graphitization degree of carbon skeleton.^[^ ^15^ ^]^ The *I*_G_/*I*_D_ values for pure‐phase Co_7_Fe_3_ alloy, Co_7_Fe_3_/Co‐600, Co_7_Fe_3_/Co‐700, and Co_7_Fe_3_/Co‐800 are 1.25, 1.37, 1.40, and 1.48, respectively, suggesting that the graphitization of carbon skeleton improves gradually with the raised pyrolysis temperature.

**Figure 2:**
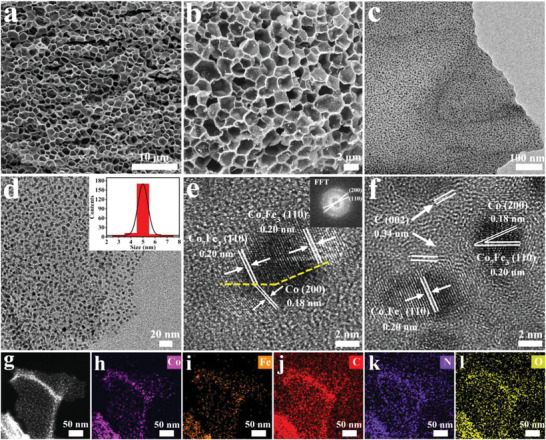
a,b) The SEM and c,d) TEM images of Co_7_Fe_3_/Co‐600 show the uniform distribution of Co_7_Fe_3_ and Co in the 3D honeycomb‐like structure; e,f) HRTEM images and Fast Fourier transform (inset) of Co_7_Fe_3_/Co‐600; g–l) The dark‐field‐scanning transmission election microscope and the corresponding element mapping distribution.

Morphology and microstructure of the sample are characterized by scanning electron microscopy (SEM) and transmission electron microscopy (TEM) images. **Figure** **2**a shows that the “beehive” is composed of hexagonal‐like holes that are interconnected by the honeycomb wall made of carbon sheets. The construction of ordered framework with open and well‐developed 3D channels is favor of large specific surface area and abundant porous structure.^[^ ^16^ ^]^ To investigate the porosity of both pure‐phase Co_7_Fe_3_ alloy and Co_7_Fe_3_/Co samples, N_2_ adsorption–desorption isotherms are further measured. As displayed in Figure S3 (Supporting Information), all the samples present an obvious hysteresis loop within the range of 0.4 < *P*/*P*_0_ < 1.0, representing a clear feature of the type‐IV isotherm of mesoporous materials.^[^ ^17^ ^]^ The surface areas and total pore volumes follow the order of Co_7_Fe_3_/Co‐600 (272.28 m^2^ g^−1^, 0.254 cm^3^ g^−1^) > pure‐phase Co_7_Fe_3_ alloy (222.02 m^2^ g^−1^, 0.228 cm^3^ g^−1^) > Co_7_Fe_3_/Co‐700 (171.15 m^2^ g^−1^, 0.151 cm^3^ g^−1^) > Co_7_Fe_3_/Co‐800 (148.03 m^2^ g^−1^, 0.127 cm^3^ g^−1^). The reduction of specific surface area and pore volume at high temperatures (700 and 800°C) should be due to the agglomeration of nanoparticles and the collapse of honeycomb skeleton. As shown in the enlarged SEM image of Co_7_Fe_3_/Co‐600 (Figure 2b), a large number of nanoparticles are homogeneously embedded within the honeycomb and remain uniformly at the micrometer scale. For comparison, Figure S4 (Supporting Information) exhibits the local magnification images of Co_7_Fe_3_ alloy, Co_7_Fe_3_/Co‐600, Co_7_Fe_3_/Co‐700, and Co_7_Fe_3_/Co‐800. It is evident that the thickness of honeycomb wall gradually increases from 60 to 130 nm. Moreover, the gain size of nanoparticles has experienced a steep increase when the temperature reaches to 700°C. These results demonstrate that the honeycomb architecture is highly dependent on thermal treatment temperature. The wettability tests of all samples are performed to investigate the interfacial molecular/ion interaction relating to water wetting. Co_7_Fe_3_/Co‐600 shows the hydrophilic nature with a very low contact angle of 8.58°, showing a significant reduction in contrast to others (Figure S5, Supporting Information). The improved surface wettability of Co_7_Fe_3_/Co‐600 can assist the kinetical adsorption/dissociation in electrocatalysis and accelerate overall water splitting.^[^ ^18^ ^]^

TEM and high‐resolution TEM (HRTEM) are used to obtain more structural features of Co_7_Fe_3_/Co‐600. Figure 2c,d shows that the honeycomb wall is composed of carbon nanosheets with attached Co_7_Fe_3_ and Co nanoparticles. It is hardly to distinguish them apart due to the similar dark spots, however, all the sphere‐like particles are homogeneous in a large scale with the uniform grain size of ≈5 nm. The formation of highly‐dispersed and ultra‐fine grain should be attributed to the features of PVP, which serves as a stabilizer to dissolve metallic ions and prevents the particle aggregation in nucleating process due to the steric hindrances.^[^ ^19^ ^]^ As revealed in Figure 2e,f, the lattice fringe spacings of 0.20 and 0.18 nm are consistent with the Co_7_Fe_3_ (110) and Co (200) planes, respectively. The obvious boundary region between Co_7_Fe_3_ and Co clarifies the formation of heterojunctions, which are surrounded by carbon layers with a distance of 0.34 nm. These tightly interfacial contacts between Co_7_Fe_3_, Co, and graphitic carbon will accelerate the electron transport in different components and be beneficial to inducing more active sites at the interfaces. The local dark‐field‐scanning transmission electron microscope and the corresponding element mapping distribution manifest the even distribution of Co, Fe, C, N, and O elements in the entire skeleton (Figure 2g–l). It is evident that both Co and Fe elements present the similar pattern, implicating that Co and Fe atoms are existed in the same compound (Co_7_Fe_3_ alloys) or closely contacted at interfaces. In addition, the doped N atoms are uniformly distributed on the carbon skeleton, which should originate from the thermal decomposition of PVP, while the existence of O atoms is attributed to the inevitable oxidation of metallic species on the catalyst surface.

**Figure 3:**
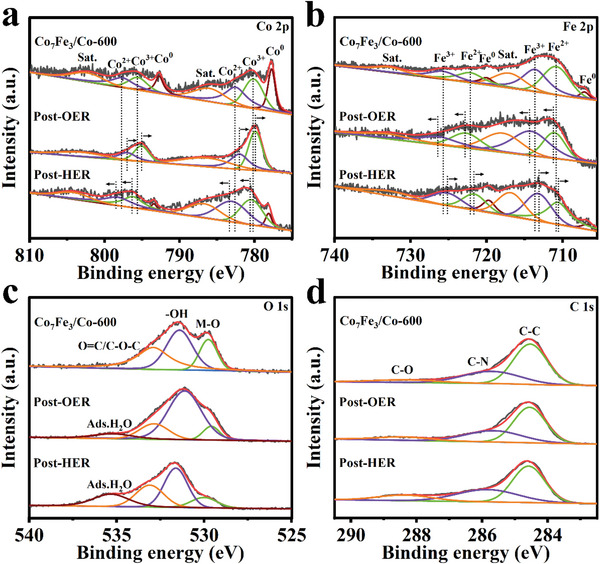
High resolution XPS spectra of a) Co 2*p*, b) Fe 2*p*, c) O 1*s*, and d) C 1*s* for Co_7_Fe_3_/Co‐600 before and after OER/HER stability tests.

X‐ray photoelectron spectroscope (XPS) is adopted to probe the surface compositions and the chemical states. The full XPS survey spectrum in Figure S6 (Supporting Information) identifies Co, Fe, C, N, and O elements, which is agree with above mentioned TEM mapping results (Figure 2h–l). The high‐resolution XPS spectrum of Co 2*p* in Co_7_Fe_3_/Co‐600 (**Figure** **3**a) shows the two peaks at 777.7 and 792.7 eV, which are attributed to zero‐valence cobalt (Co^0^) in both metallic Co and Co_7_Fe_3_ alloy.^[^ ^8b^ ^]^ The peaks at 782.5 and 797.6 eV can be assigned to Co^2+^ in Co 2*p*_3/2_ and Co 2*p*_1/2_, while the peaks located at 780.1 and 795.6 eV correspond to Co^3+^ of Co 2*p*_3/2_ and Co 2*p*_1/2_, respectively.^[^ ^20^ ^]^ These results suggest that the Co species of Co_7_Fe_3_/Co‐600 exist in the forms of Co^0^, Co^2+^, and Co^3+^ simultaneously. The presence of high‐valence sate Co species is resulted from the inevitable surface oxidation. Similarly, the high‐resolution spectrum of Fe 2*p* (Figure 3b) shows that the peaks at 707.1 and 720.0 eV correspond to zero‐valence iron (Fe^0^) in alloy.^[^ ^21^ ^]^ The peaks at 710.8 and 713.6 eV are ascribed to Fe^2+^ and Fe^3+^ in Fe 2*p*_3/2_, and a pair of peaks located at 722.1 and 725.7 eV represent Fe^2+^ and Fe^3+^ in Fe 2*p*_1/2_, respectively.^[^ ^22^ ^]^ As reported previously, the Co^3+^/Fe^3+^ species originated from Co^0^/Fe^0^ (Co^2+^/Fe^2+^) act as the main active sites for OER due to the formation of key active species (MOOH).^[^ ^23^ ^]^ Focusing on O 1*s* spectrum, it exhibits the peaks at 529.8, 531.4, and 532.9 eV, which are related to M—O, —OH, and O=C/C—O—C, respectively (Figure 3c). Amongst, the presence of M—O group verifies the occurrence of oxidation on the metal surface.^[^ ^24^ ^]^ The O‐containing groups are advantageous to hydrophilicity, thus assisting the electrocatalytic kinetics.^[^ ^25^ ^]^ In Figure 3d, the dominant peak at 284.6 eV originates from the *sp* ^2^ hybrid graphite, indicating the formation of graphitic carbon in as‐prepared catalyst. Both graphitic N and pyridinic N are also detected in XPS spectrum of the N 1*s* (Figure S7, Supporting Information).

### 2.2 Electrocatalytic Performance Evaluation

#### 2.2.1 OER Performance

**Figure 4:**
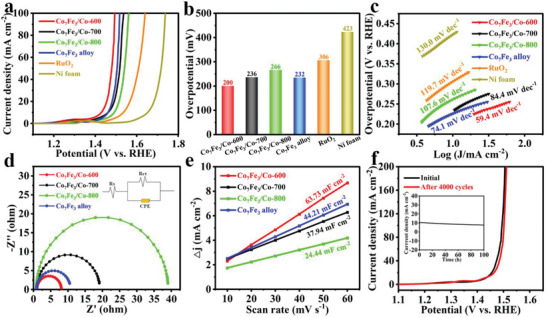
a) LSV curves; b) Overpotential at 10 mA cm^−2^; c) Tafel slopes of pure‐phase Co_7_Fe_3_ alloy, Co_7_Fe_3_/Co‐600, Co_7_Fe_3_/Co‐700, Co_7_Fe_3_/Co‐800, Ni foam, and RuO_2_; d) EIS plots for Co_7_Fe_3_ alloy, Co_7_Fe_3_/Co‐600, Co_7_Fe_3_/Co‐700, and Co_7_Fe_3_/Co‐800. The inset in (d) is the equivalent circuit; e) The double layer capacity (*C*_dl_) value of different samples; f) LSV curves of Co_7_Fe_3_/Co‐600 before and after 4000 CV cycles. Inset: the current–time (*i–t*) curve of Co_7_Fe_3_/Co‐600 for 100 h.

The electrocatalytic performances of Co_7_Fe_3_/Co heterostructure and pure‐phase Co_7_Fe_3_ alloy toward OER are examined in 1.0 m KOH solution by a typical three‐electrode system. The bare Ni form and RuO_2_‐loaded electrodes are used for comparisons. All linear sweep voltammetry (LSV) data for OER are measured after *iR* compensation to fairly compare their catalytic activities. As shown in **Figure** **4**a,b, to achieve a current density of 10 mA cm^−2^, all the as‐made catalysts are evaluated in this study, and the Co_7_Fe_3_/Co‐600 hybrid requires the lowest overpotential of 200 mV, which is much superior to those of Co_7_Fe_3_/Co‐700 (236 mV), Co_7_Fe_3_/Co‐800 (266 mV), Co_7_Fe_3_ alloy (232 mV), RuO_2_ (306 mV), and Ni foam (423 mV). Notably, the OER activity of Co_7_Fe_3_/Co‐600 is also superior to most recently‐reported alloys‐based catalysts in alkaline media (Table S1, Supporting Information). The significantly enhanced electrocatalytic activity on Co_7_Fe_3_/Co‐600 is chiefly attributed to the synergetic optimization of active components. On the one hand, PVP‐stabilized temperature‐program carbonization endows the homogeneous and ultra‐fine nanoparticles in a well‐supported 3D graphitic carbon framework. This intentionally‐designed structure allows active species to be fully exposed to electrolyte, thus driving a high OER activity. On the other hand, the formed Co_7_Fe_3_/Co heterojunctions should eagerly induce the interfacial electronic effects, which enable the catalytic surface favorable for specific reactants and promote the generation of highly active species (MOOH), thus augmenting the catalytic activity.^[^ ^26^ ^]^ Following, the electrochemical dynamics at the catalyst/electrolyte interface are explored to understand the OER activity on Co_7_Fe_3_/Co‐600. As shown in Figure 4c, Co_7_Fe_3_/Co‐600 catalyst has a smaller Tafel slope of 59.4 mV dec^−1^ than those of Co_7_Fe_3_/Co‐700 (84.4 mV dec^−1^), Co_7_Fe_3_/Co‐800 (107.6 mV dec^−1^), Co_7_Fe_3_ alloy (74.1 mV dec^−1^), RuO_2_ (119.7 mV dec^−1^), and Ni foam (130.0 mV dec^−1^), indicating that Co_7_Fe_3_/Co‐600 catalyst can obtain a faster charge transfer across the electrocatalytic interface at lower potentials, which is further conducive to improving the reaction kinetics during OER process.^[^ ^27^ ^]^

Electrochemical impedance spectroscopy (EIS) results further show that Co_7_Fe_3_/Co‐600 has the smallest semicircular diameter, indicating its good conductivity and quick charge transfer for OER (Figure 4d). Electrochemical active surface area (ECSA) is reflected by the double‐layer capacitance (*C*_dl_) from cyclic voltammetry (CV) in a non‐Faradaic region of 1.0–1.1 V (vs. RHE) and with the incrementally scanning rates of 10–60 mV s^−1^ (Figure S8, Supporting Information). As shown in Figure 4e, the *C*_dl_ values of Co_7_Fe_3_/Co‐600, Co_7_Fe_3_/Co‐700, Co_7_Fe_3_/Co‐800, and pure‐phase Co_7_Fe_3_ alloy are 63.73, 37.94, 24.44, and 44.21 mF cm^−2^, respectively. Accordingly, the ECSA values of Co_7_Fe_3_/Co‐600, Co_7_Fe_3_/Co‐700, Co_7_Fe_3_/Co‐800, and pure‐phase Co_7_Fe_3_ alloy are 1593.3, 948.5, 611.0, and 1105.3 cm^2^, respectively. The large ECSA of Co_7_Fe_3_/Co‐600 is ascribed to its highly porous and open structure with a high exposure of active sites. The long‐time stability of electrocatalysts is a key critical criterion for practical energy conversion systems. Thus, the LSV curve of Co_7_Fe_3_/Co‐600 cycles is measured after 4000 CV cycles, which demonstrates that the current decay is negligible (Figure 4f). Moreover, Co_7_Fe_3_/Co‐600 catalyst can maintain a stable current density ≈10 mA cm^−2^ without a remarkable descent after continuous electrolysis for 100 h, confirming its excellent durability in 1.0 m KOH (inset in Figure 4f).

For a deep understanding of electrocatalytic mechanisms, XPS and in situ XRD tests are conducted to investigate surface valence and structure/phase changes during OER. By comparing the XPS spectra (Figure 3a), it is found that Co^0^ in Co_7_Fe_3_/Co‐600 is almost entirely converted to Co ionic valence state after OER. Furthermore, both Co 2*p*_1/2_ and Co 2*p*_3/2_ XPS peaks become narrower and shift ≈0.2–0.6 eV to lower binding energies, which can be attributed to the oxidation of Co^2+^ to Co^3+^ after OER.^[^ ^28^ ^]^ Similar phenomenon is detected for Fe 2*p* peaks that shift ≈0.3–0.6 eV to higher binding energies, suggesting the oxidation of Fe^2+^ to Fe^3+^ (Figure 3b).^[^ ^29^ ^]^ Correspondingly, the peak area percentages of Co^3+^ and Fe^3+^, along with the ratios of both Co^3+^/Co^2+^ and Fe^3+^/Fe^2+^ obviously increase after OER (Tables S2 and S3, Supporting Information). These results support the formation of cobalt/iron oxyhydroxide (CoOOH/FeOOH), which are served as active species for the OER intermediate reaction (MO + OH^−^→MOOH + e^−^).^[^ ^30^ ^]^ The O 1*s* XPS spectra in Figure 3c further reveal —OH peak shifts slightly to a lower energy after OER, evidencing the formation of —OO,^[^ ^31^ ^]^ and the peak area integration increases from 42.3% to 65.4% (Table S4, Supporting Information), once again suggesting that the metallic species are hydroxylated and form surface hydrated oxides or (oxy)hydroxides after OER test.^[^ ^32^ ^]^ In addition, the C1*s* spectrum does not change significantly, indicating that the carbon carrier is quite stable during OER in alkaline media. In situ XRD can provide the real‐time monitoring of phase changes in catalyst under realistic electrochemical working conditions. The schematic diagram and XRD patterns are shown in Figure S9 (Supporting Information). As displayed in Figure S9b (Supporting Information), new diffraction peaks are detected at ≈35.27°, 38.23°, and 41.44°, which are well indexed to the (130), (040), and (200) lattice planes of CoOOH (JCPDS No. 26‐0480), respectively. Moreover, the peaks at ≈39.08° and 59.02° correspond to the (200) and (151) lattice planes of FeOOH (JCPDS No. 29‐0713), respectively. These results have well recognized the surface reconstruction of Co_7_Fe_3_/Co‐600 catalyst during OER through phase transitions into CoOOH and FeOOH. This structure evolution on the catalyst surface will generate more favorable and stable active sites for enhanced OER performance.^[^ ^33^ ^]^

#### 2.2.2 HER Performance

**Figure 5:**
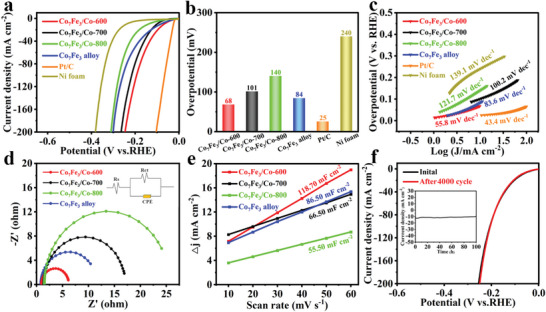
a) LSV curves; b) Overpotentials at 10 mA cm^−2^; c) Tafel slopes and d) EIS plots of pure‐phase Co_7_Fe_3_ alloy, Co_7_Fe_3_/Co‐600, Co_7_Fe_3_/Co‐700, Co_7_Fe_3_/Co‐800, Ni foam, and Pt/C (The inset in d) is the equivalent circuit); e) The *C*_dl_ values of different catalysts; f) Polarization curves of Co_7_Fe_3_/Co‐600 before and after 4000 CV cycles. Inset: the current–time (*i–t*) curve of Co_7_Fe_3_/Co‐600 for 100 h.

The electrocatalytic activities for HER are also examined in 1.0 m KOH electrolyte. As shown in **Figure** **5**a,b, Co_7_Fe_3_/Co‐600 catalyst exhibits a much lower *η*_10_ value (68 mV) than those of Co_7_Fe_3_/Co‐700 (101 mV), Co_7_Fe_3_/Co‐800 (140 mV) and pure‐phase Co_7_Fe_3_ alloy (84 mV), while the commercial Pt/C catalyst shows the highest activity toward HER (25 mV). It indicates that the structure‐optimized Co_7_Fe_3_/Co heterojunctions can obtain the outstanding HER activity. Furthermore, based on the BET and SEM results, it is deduced that the excellent catalytic activity of Co_7_Fe_3_/Co‐600 is improved by the increased number of exposed active sites (compared with Co_7_Fe_3_/Co‐700 and Co_7_Fe_3_/Co‐800). However, by comparing with pure‐phased Co_7_Fe_3_ alloy, the promoted performance of Co_7_Fe_3_/Co‐600 is attributed to the assisting role of metallic Co, which induces the electronic redistribution and provides the additional active sites, thus promoting the intrinsic catalytic activity. Tafel analysis is performed to evaluate the reaction kinetics. As presented in Figure 5c, apart from the smallest Tafel slope (43.4 mV dec^−1^) for commercial Pt/C catalyst, the Tafel slope for Co_7_Fe_3_/Co‐600 is 55.8 mV dec^−1^, which is much lower than those of pure‐phase Co_7_Fe_3_ alloy (83.6 mV dec^−1^), Co_7_Fe_3_/Co‐700 (100.2 mV dec^−1^), and Co_7_Fe_3_/Co‐800 (121.7 mV dec^−1^). It is worth noting that although the HER pathways on Co_7_Fe_3_/Co‐600 hybrid and Co_7_Fe_3_ alloy still follow the Volmer–Heyrovsky mechanism,^[^ ^34^ ^]^ the incorporation of metallic Co to alloy has indeed led to a remarkable decrease in Tafel slope, implying a more favorable reaction kinetics on Co_7_Fe_3_/Co‐600 electrode. The enhanced catalytic activity is also verified by LSV curves normalized with Brunauer–Emmett–Teller (BET) surface areas and the loading weight of catalyst on electrode (Figure S10, Supporting Information). Furthermore, the charge transfer resistance (*R*_ct_) and *C*_dl_ values are obtained to explore the catalytic kinetics (Figure 5d,e). Co_7_Fe_3_/Co‐600 shows a higher *C*_dl_ (ECSA) value and a lower charge transfer resistance than other as‐prepared catalysts (Figure S11, Supporting Information), indicating that it has a faster charge transfer process and exposes more available active sites. Notably, Figure 5f reveals the outstanding durability of Co_7_Fe_3_/Co‐600 toward HER in alkaline media, in which the LSV curve after 4000 CV cycles almost completely overlaps with the initial one, and the *i–t* curve shows a negligible decline (0.6%) after 100 h of tests (inset of Figure 5f). The superiority of Co_7_Fe_3_/Co‐600 to most of the reported alloying catalyst is also displayed in Table S1 (Supporting Information). After HER stability test, it is found that the deconvoluted peaks for Co 2*p* show a positive shift (0.3–0.7 eV), and the characteristic peaks of Fe 2*p* display a negative shift (0.3–0.5 eV). This should correspond to the transformation of Co^3+^ to Co^2+^ and Fe^3+^ to Fe^2+^, revealing that the high valence metal species have been reduced into lower valence states that serve as actual active phase for HER.^[^ ^35^ ^]^

The compositional and structural stability of Co_7_Fe_3_/Co‐600 after OER and HER are further explored by XRD and TEM. As shown in Figure S12 (Supporting Information), the characteristic diffraction peaks of both Co_7_Fe_3_ and Co remained after OER and HER tests, indicating the crystalline matrix of Co_7_Fe_3_/Co‐600 is well preserved. Notably, a set of week peaks for CoOOH/FeOOH species are observed after OER, which is completely consistent with the results of in situ XRD. It further confirms the Co_7_Fe_3_/Co‐600 surface sites undergo the dynamic reconstruction and transform into real reactive species.^[^ ^36^ ^]^ As shown in Figure S13 (Supporting Information), the morphology of Co_7_Fe_3_/Co‐600 remains almost unchanged after the long‐term electrochemical corrosion, in which nanoparticles are still uniformly dispersed on the carbon sheets in a high density without obvious corrosion/aggregation, and the heterogeneous interface is visible clearly, confirming the excellent structural stability of Co_7_Fe_3_/Co‐600.

### 2.3 Density Functional Theory (DFT) Calculations

**Figure 6:**
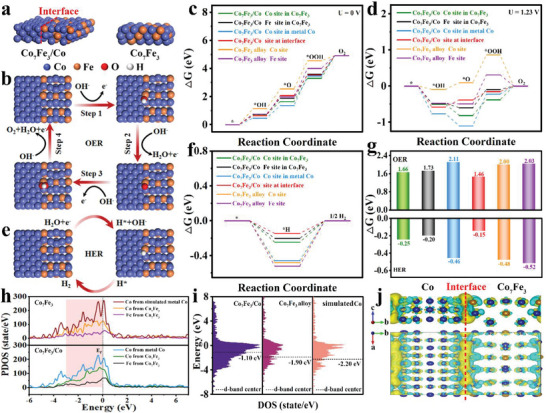
a) Theoretical models of Co_7_Fe_3_/Co and pure‐phase Co_7_Fe_3_; b) The optimized adsorption configurations of oxygen intermediates (*OH, *O, and *OOH) on the interface; Free energy diagrams for OER on Co_7_Fe_3_/Co and Co_7_Fe_3_ c) at zero potential (*U* = 0) and d) equilibrium potential (*U* = 1.23 V); e) The theoretical model for *H adsorbed on Co_7_Fe_3_/Co; f) Free energy diagram for HER on Co_7_Fe_3_/Co and Co_7_Fe_3_; g) Calculated *OOH and *H adsorption energy on Co_7_Fe_3_/Co and Co_7_Fe_3_; h) The partial density of states (PDOS) of surface models (Fermi level is denoted by dashed lines) and i) *d*‐band centers; j) Differential charge density at the interface (The yellow and blue represent the charge accumulation and depletion, respectively).

Determination of intrinsic active sites of a catalyst is always important for understanding the catalyst feature. Although it is quite evident that the optimized Co_7_Fe_3_/Co hybrid exhibits a much‐improved electrocatalytic activity than pure‐phase Co_7_Fe_3_ alloy, whether for OER or HER, it is still unclear where the inherent active center is and which active sites predominately contribute to the OER or HER. To answer these questions, density functional theory (DFT) calculations are performed to quantitatively illuminate the origin of Co_7_Fe_3_/Co hybrid for enhanced bifunctional activity (calculation details are provided in Supporting Information). **Figure** **6**a exhibits the models of Co_7_Fe_3_/Co hybrid and pure‐phase Co_7_Fe_3_ alloy, which are built based on the findings of XRD and TEM results, as well as the DFT optimization. Specifically, the four‐electron mechanism that proceeds with the adsorption of intermediates (*OH, *O, and *OOH) on different sites is proposed to study the OER process in alkaline electrolyte, then the optimized models of various possible adsorption sites are displayed in Figure 6b and Figures S14 and S15 (Supporting Information).^[^ ^37^ ^]^ The Gibbs free energy change (Δ*G*) is employed to describe the affinity responsible for model molecules on catalyst surface. The obtained Δ*G* diagrams in Figure 6c,d and Table S5 (Supporting Information) disclose that the conversion of *O into *OOH (*O + OH^−^ → *OOH + e^−^) is the rate‐determining step (RDS). In the case of pure‐phase Co_7_Fe_3_ alloy, the Δ*G*_rds_ values are 2.00 and 2.03 eV for Co site and Fe site, respectively (Figure 6g). In contrast, a dramatic decrease is found for Co site (1.66 eV) and Fe site (1.73 eV) in Co_7_Fe_3_ of Co_7_Fe_3_/Co hybrid. It indicates that the Co and Fe sites in alloy are the active OER sites, and their reaction kinetic is accelerated after the introduction of metallic Co. Notably, the Δ*G*_rds_ value further decreases to 1.46 eV at the interface site of Co_7_Fe_3_/Co, which demonstrates that the most contribution to promote OER activity should come from the heterointerfaces. On the other hand, the single metallic Co site in Co_7_Fe_3_/Co shows a large energy barrier (2.11 eV). Such result reveals that the OER process on a single metallic Co is sluggish, however, the incorporation of metallic Co can optimize the adsorption energy on nearby heterointerface and long‐range Co_7_Fe_3_ sites that will effectively decrease the reaction barrier of Co_7_Fe_3_/Co. Similarly, these crystal models are implemented to study the HER process (Figure 6e; Figures S16 and S17, Supporting Information). The Gibbs free energy change of hydrogen adsorption (∆*G*_H*_) is used as a main indicator for HER.^[^ ^38^ ^]^ The closer the ∆*G*_H*_ is to zero, the better the hydrogen adsorption and desorption kinetics will be.^[^ ^39^ ^]^ As shown in Figure 6f,g, the Δ*G*_H*_ value at Co_7_Fe_3_/Co interface site is −0.15 eV, which is closer to ∆*G*_H*_ = 0 than other active sites and undoubtedly considered as the main active center for HER. In addition, in the Co_7_Fe_3_/Co hybrid, the Δ*G*_rds_ values are −0.25 and −0.20 eV for the Co and Fe sites, respectively, which are close to the Δ*G*_H*_ value at the interface site, so they can also be considered as secondary active HER sites.

To further understand the assisting effect of metallic Co on the improved intrinsic activity, density of states (DOS) and differential charge density (DCD) calculations are performed. It is well known that the interfacial chemical interaction often leads to the changes of local electronic coordination of the near‐interface species. The electron transfer and rearrangement occur due to their differences in Fermi energy levels. Electrons may be shared equally or unequally between directly connected active species, resulting in electron transfer in heterojunctions or electron delocalization.^[^ ^40^ ^]^ As shown in Figure 6h, compared with the theoretically simulated Co (red line), the electron density of the single metallic Co (blue line) in Co_7_Fe_3_/Co at Fermi level (*E*_f_ = 0 eV) is greatly reduced. In contrast, the electron densities corresponding to Co (green line) and Fe (black line) in Co_7_Fe_3_ of Co_7_Fe_3_/Co are slightly larger than those of Co (yellow line) and Fe (pink line) in pure‐phase Co_7_Fe_3_. This result indicates that the electrons on metallic Co are exploited and transfer to Co_7_Fe_3_ alloy. Furthermore, the marked region in Figure 6h reveals the electronic delocalization on Co_7_Fe_3_, which mainly originates from the electron transfer across the Co_7_Fe_3_/Co interface. This electronic delocalization at the nanometer scale will lead to the formation of additional charged species, which may provide more favorable adsorption sites for key intermediates and stable coordination environments for electrocatalysis.^[^ ^41^ ^]^ The *d*‐band center (*E*_d_) is also an important descriptor for the affinity between catalysts and model molecules. Figure 6i gives the calculations of *d*‐band center (*E*_d_), by which the *E*_d_ for hetero‐structured Co_7_Fe_3_/Co (−1.10 eV) is the closest to Fermi energy level, revealing the most favorable affinity for OER/HER intermediates.^[^ ^42^ ^]^ Furthermore, Figure 6j presents the DCD spectrum to unveil the electron distribution in Co_7_Fe_3_/Co. It is found that the construction of heterostructure enables more electrons on metallic Co to be deprived and transferred to nearby interface and long‐range Co_7_Fe_3_ layer, which eagerly constructs electron‐rich interface over Co_7_Fe_3_/Co and creates more effective active centers for both OER and HER.

### 2.4 Performance of Overall Water Splitting on Co7Fe3/Co‐600 Catalyst

**Figure 7:**
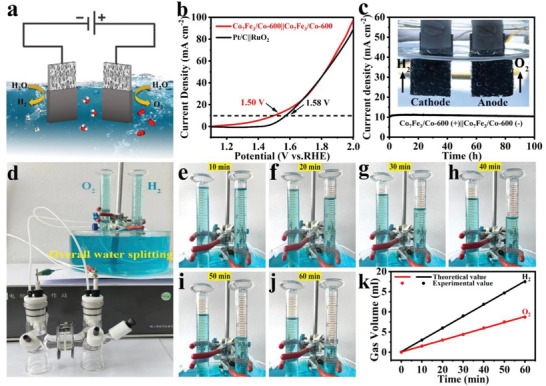
a) Schematic diagram of the overall water splitting system; b) LSV curves of the overall water splitting in 1.0 m KOH in a two‐electrode system; c) *I–t* curve of Co_7_Fe_3_/Co‐600 for 100 h of test in 1.0 m KOH (The inset shows the bubbles of H_2_ and O_2_ generated from electrodes); d) A drainage setup for collecting H_2_ and O_2_ produced; e–j) Enlarged digital images of the measuring gas quantity generated at 10 min intervals for a total of 60 min; k) Amounts of H_2_ and O_2_ as a function of time.

To demonstrate the application potential of Co_7_Fe_3_/Co‐600 in overall water splitting, a two‐electrode electrolyzer is assembled, adopting Co_7_Fe_3_/Co‐600 electrode as both the anode and cathode (**Figure** **7**a). As shown in Figure 7b, compared with a high voltage of 1.58 V on Pt/C||RuO_2_, Co_7_Fe_3_/Co‐600||Co_7_Fe_3_/Co‐600 only requires a cell voltage of 1.50 V to achieve a current density of 10 mA cm^−2^ to split water into molecular hydrogen and oxygen. This result also shows the significant advantages over most of the reported alloying catalysts (Table S1, Supporting Information). Moreover, the stability is tested at a constant cell potential of 1.50 V over 100 h. As shown in Figure 7c, the cell remains ≈99.1% of the initial current density after 100 h of continuous reaction. The exceptional stability of Co_7_Fe_3_/Co‐600 catalyst for overall water splitting should be mainly attributed to the following factors: i) Co_7_Fe_3_/Co nanoparticles are normally dispersed in PVP matrices acting as the capping agent to avoid the aggregation; ii) Co_7_Fe_3_/Co catalyst generates beneficial and stable active surface phases under the applied voltage, leading to the enhancement of catalytic stability at a given overvoltage; iii) Electronic redistribution after coupling Co with Co_7_Fe_3_ changes the *d*‐band center position of Co_7_Fe_3_/Co and provides additional active sites at the interfaces to essentially improve the activity and stability.^[^ ^43^ ^]^ In addition, the good hydrophilicity helps Co_7_Fe_3_/Co‐600 to energetically accelerate the diffusion of electrolyte and rapidly divert the adhered H_2_/O_2_ bubbles away from the electrode to greatly decrease mechanical shock during long cycles.^[^ ^44^ ^]^ To evaluate overall water splitting efficiency, the H_2_ and O_2_ gases generated from the electrolyzer can be collected and analyzed by the drainage method (Figure 7d). Figure 7e–j record the levels of H_2_ and O_2_ at 10 min intervals for a total of 60 min. Moreover, the gas yield‐time relationship in Figure 7k confirms the volume ratio of H_2_ and O_2_ is almost 2: 1, meaning the Faraday efficiency of overall water splitting attains 100%. Moreover, the electrolyzer can be driven by a solar cell at a low voltage of 1.49 V, implying its practical application potential for coupling with renewable energy (Figure S18 and Movie S1, Supporting Information).

## 3 Conclusion

In summary, we have successfully constructed a 3D honeycomb‐like graphitic carbon supported Co_7_Fe_3_/Co heterostructure, by which synergistic structure and electron modulating interfaces are formed, thus simultaneously promoting the catalytic kinetic, activity and stability. Specifically, i) the high‐dispersed and ultra‐fine nanoparticles embedding in an open carbon framework allow active species to be fully exposed; ii) The porous structure, large specific surface area and hydrophilicity facilitate the electrolyte diffusion and transportation; iii) The graphitized carbon substrate improves the conductivity and prevents the dissolution/aggregation of particles in electrcatalysis; iv) The electronic exploitation and diversion from metallic Co to interface and long‐range Co_7_Fe_3_ alloy induce the electron‐rich state over interface and the delocalized electronic state on Co_7_Fe_3_, which contribute to the optimization of intermediates adsorption free energy and thus promote the intrinsic catalytic activity. This work aims to understand the origin of alloy/single metal heterojunction for highly effective water splitting, which opens a new path for rational design of bifunctional catalysts.

## 4 Experimental Section

### Preparation of Co_7_Fe_3_/Co Heterojunctions

Typically, PVP (1.0 g) was dissolved into 30 mL of DI water under magnetic stirring at room temperature (25°C). Then Co(NO_3_)_2_·6H_2_O (1.05 g) and Fe(NO_3_)_3_·9H_2_O (0.45 g) were simultaneously added to the above PVP solution and followed by vigorous stirring for 5 h. After that, the above mixture was dried overnight at 95°C to form the PVP‐Co^2+^‐Fe^3+^ precursor, which was then carbonized at different temperatures (500–800°C) for 1 h under the N_2_ atmosphere with a ramp rate of 5°C min^−1^. The pure‐phase Co_7_Fe_3_ was obtained at 500°C and named as Co_7_Fe_3_ alloy for short. A series of Co_7_Fe_3_/Co hybrids were obtained at 600, 700, and 800°C, which were denoted as Co_7_Fe_3_/Co‐600, Co_7_Fe_3_/Co‐700, and Co_7_Fe_3_/Co‐800, respectively. Material characterization methods, including XRD, SEM, TEM, XPS, TG‐DSC, Raman, N_2_ adsorption adsorption/desorption isotherms, contact angle, and DFT calculations were described in the “Supporting Information”. Electrochemical tests were also provided in the “Supporting Information”, including CV, LSV, EIS, *C*_dl_, ECSA, CA, FE, etc.

### Statistical Analysis

Pre‐processing of the data was carried out in Excel and the final data were statistically analyzed and plotted using Origin (version 2021) software. Electrochemical test data were converted with reference to reversible hydrogen electrodes (RHE). The results were performed in triplicates and repeated three or more times to obtain an average.

## Conflict of Interest

The authors declare no conflict of interest.

## Supporting information

Supporting Information

Supplemental Movie 1

## Data Availability

The data that support the findings of this study are available from the corresponding author upon reasonable request.
