# Supplementary material for: Electronic‐State Modulation of Metallic Co‐Assisted Co7Fe3 Alloy Heterostructure for Highly Efficient and Stable Overall Water Splitting
Source: Adv Sci (Weinh). 2023 May 23;10(22):2301961. doi: 10.1002/advs.202301961 (PMC10401179; doi:10.1002/advs.202301961)
Supplement: Supplementary file 1 — Supporting Information [file ADVS-10-2301961-s001.pdf]

ADVANCED SCIENCE
Open Access

Supporting Information

for *Adv. Sci.*, DOI 10.1002/advs.202301961

Electronic-State Modulation of Metallic Co-Assisted Co_7Fe_3 Alloy Heterostructure for Highly Efficient and Stable Overall Water Splitting

Xinyu Wang, Xiaoqin Xu, Yao Nie, Ruihong Wang and Jinlong Zou**

Electronic-State Modulation of Metallic Co-assisted Co_7Fe_3 Alloy Heterostructure for Highly Efficient and Stable Overall Water Splitting

Xinyu Wang[a], *Xiaoqin Xu*[a], *Yao Nie*[a], *Ruihong Wang*[a,]*, *and Jinlong Zou*[a,]*

[a] Key Laboratory of Functional Inorganic Material Chemistry, Ministry of Education of the People's Republic of China, School of Chemistry and Materials Science, Heilongjiang University, Harbin, 150080, China.

Corresponding author (s):

* Ruihong Wang, Jinlong Zou.

Xuefu Road 74#, Nangang District, Harbin, 150080, China.

E-mail: wangruihong@hlju.edu.cn (R. H. Wang); zoujinlong@hlju.edu.cn (J. L. Zou).

1. Experimental methods

1.1 Material Characterizations

Herein, the structure, morphology and compositions of the as-prepared materials were characterized by X-ray diffraction (XRD), X-ray photoelectron spectroscopy (XPS), N_2 adsorption/desorption isotherms, scanning electron microscope (SEM), transmission electron microscopy (TEM) and elemental mappings (energy-dispersive X-ray spectroscopy, EDS). X-ray diffraction (XRD) patterns were performed with an X-ray diffractometer (XRD, Bruker D8 Advanced) with an accelerating voltage of 40 kV. The Raman data were collected using a Raman spectrometer (Jobin Yvon HR800). Thermogravimetric (TG) and Differential Scanning Calorimetry (DSC) analysis were performed using a TA Q600 thermal analyzer under a stream of N_2 with a heating rate of 10 °C min^{-1}. The morphologies of the synthesized samples were studied by SEM (Philips XL-30-ESEM-FEG, 5-20 kV). Transmission electron microscopy (TEM), high-resolution TEM (HRTEM) and energy-dispersive spectroscopy measurement (EDS) were implemented on TEM JEOL JEM-3010. The chemical state and surface composition were investigated by the X-ray photoelectron spectroscopy (XPS, VG ESCALAB MK II). The Brunauer-Emmett-Teller (BET) surface area of the product was measured by using N_2 adsorption-desorption (TriStar II 3020); the sample was dried under vacuum at 150 °C for 5 h before measurement.

1.2 Electrochemical measurements

All the electrochemical measurements were carried out at the CHI 760E electrochemical workstation with a typical three-electrode system and the overall water splitting test was carried out in a two-electrode configuration under 1.0 M KOH electrolyte at room temperature (25 °C). In the three-electrode electrochemical system, electrocatalyst-loaded on Ni foam (1 cm^2) was used as the working electrode, mercuric oxide electrode (MOE) and graphite rod were used as reference electrode and the counter electrode, respectively. The catalyst ink was prepared by 5 mg of catalyst, 1 mg carbon black and 30 μL of Nafion (5 wt%) were add to water/ethanol (1 mL, 1: 1 (v/v))

solvent mixture and the mixture was completely uniform. Subsequently, the catalyst ink was coated onto a piece of Ni foam (1.0×1.0 cm^2) and then drying in the oven at 60 °C overnight for alkaline OER and HER tests. The mass loading of catalyst was approximately 5 mg cm^{-2}. Before coating, the Ni foam was washed with acetone and HCl (2.0 M) for (immersion time: 15 min per solvent) to remove oily substances, and then deionized water was used to ultrasonically rinse acid and other organic substances. Following, dry overnight in a 60 °C oven for later use. For comparison, RuO_2-loaded electrode and Pt/C-loaded electrode were prepared by the same method. Commercial RuO_2 and 20 wt.% Pt/C catalysts are purchased from Alfa Aesar and Johnson Matthey Fuel Cells, respectively. Before performing the measurements, 20 cycles of cyclic voltammetry (CV) scans were performed to activate the electrocatalyst. Linear sweep voltammogram (LSV) was measured from 0 V to 1.0 V and -0.8 V to -1.8 V vs MOE at a scan rate of 5 mV s^{-1} and 2 mV s^{-1}, respectively, for OER and HER. For each test, the catalyst ink was evenly coated on the foam nickel substrate, and the final result (LSV) was obtained by taking the average of three sets of parallel tests. In all measurements, all potentials were referenced to reversible hydrogen electrode (RHE) based on the Nernst equation: $E_{RHE} = E_{MOE} + 0.059 \times pH + 0.098$. All the linear scan voltammetry (LSV) curves were iR compensated with a compensation level of 90 %. The Tafel plots of as-prepared catalysts were carried out from the corresponding LSV data using the following formula: $\eta = a + b \times \log j$, where a, η, b and j represent the corresponding constant, overpotential ($\eta = E_{RHE}-1.23$ for OER and $\eta = 0-E_{RHE}$ for HER), Tafel slope and current density, respectively. From the below formula, it could be seen that the slope of the Tafel plot was inversely related to the charge transfer coefficient (α), which meant that the lower the Tafel slope, the faster the charge transfer across the electrocatalytic interface.[1]

$$\text{Slope } (d \log j / d\eta) \text{ for cathodic reaction} = (2.303RT/-\alpha_C nF) \qquad (1)$$

$$\text{Slope } (d \log j / d\eta) \text{ for anodic reaction} = (2.303RT/-\alpha_A nF) \qquad (2)$$

where α_C and α_A were the charge transfer coefficients for the cathodic and anodic reactions,

respectively, n was the number of electrons transferred, F was the Faraday constant (96500 C), R was the ideal gas constant, T was the absolute temperature in K and η was the overpotential.

The electrochemically active surface area (ECSA) values were estimated by the electrochemical double layer capacitance (C_{dl}) determined by cyclic voltammetry (CV) curves performed in the non-Faradaic regions at scan rates of 10-60 mV s^{-1}. Electrochemical impedance spectroscopy (EIS) studies were carried out in the faradaic region to compare the R_{ct}. The stability of the catalysts was evaluated by performing CV cycles at a scan rate of 50 mV s^{-1} and chronoamperometry measurements at a static overpotential. The electrochemical active surface area (ECSA), which could be calculated by using the following equation:[2]

$$ECSA= C_{dl} / (40\ \mu F\ cm^{-2}\ per\ cm^2)$$

where 40 μF cm^{-2} was a constant to convert capacitance to ECSA. The specific capacitance was converted into an ECSA using the specific capacitance value for a flat standard with 1 cm^2 of real surface area.

To calculate the faradaic efficiency (FE), the generation of H_2 and O_2 generated was collected via a simple drainage method. In the two-electrode cell, constant potential electrolysis was performed at 43 mA cm^{-2} for 60 minutes and the volume of gas evolution was determined every 600 s. The theoretical H_2/O_2amount was calculated via the following equation:[3]

$$n(H_2) = Q/zF$$

$$n(O_2) = Q/zF$$

where *n* (H_2), z and Q represent the number of moles of hydrogen produced, the number of electron transfer (for HER is 2 and OER is 4), the charge passed through the electrodes, respectively. F is the Faraday constant (96500 C mol^{-1}).

1.3 In-situ X-ray diffraction (in-situ XRD) measurement

In-situ XRD was performed on a Rigaku D/max XRD diffractometer using a two-electrode electrolytic system with Co_7Fe_3/Co-600 as the working anode and Pt sheet as the cathode. OER (electrolysis cell) was driven by an external battery in 1.0 M KOH electrolyte. The changes of crystalline phases of the Co_7Fe_3/Co-600 electrode were recorded at certain intervals.

1.4 Computational details

All the calculations were performed in the framework of the density functional theory with the projector augmented plane-wave method, as implemented in the Vienna ab initio simulation package.[4] The generalized gradient approximation proposed by Perdew, Burke, and Ernzerhof was selected for the exchange-correlation potential.[5] The long-range van der Waals interaction was described by the DFT-D3 approach.[6] The cut-off energy for plane wave was set to 450 eV. The energy criterion was set to 10^{-5} eV in iterative solution of the Kohn-Sham equation. A vacuum layer of 15 Å was added perpendicularly to the sheet to avoid artificial interaction between periodic images. The Brillouin zone integration was performed using a 2×2×1 k-mesh. All the structures were relaxed until the residual forces on the atoms had declined to less than 0.03 eV/Å. The free energy changes (ΔG) of reaction intermediates could be calculated by the following equation:

$$\Delta G = \Delta E + \Delta E_{ZPE} - T\Delta S$$

where ΔE was the adsorption energy on the cluster surface from DFT calculations. The ΔE_{ZPE} and ΔS were the difference for the zero-point energy and entropy, respectively. The zero-point energy and entropy were calculated at the standard conditions corresponding to the pressure of 101325 Pa (~1 bar) of H_2 at the temperature of 298.15 K.

2. Results and discussion

Table S1. Comparisons of OER, HER and overall water splitting performance of Co_7Fe_3/Co-600 with other reported Co-based alloys electrocatalysts in alkaline electrolyte.

**Catalysts**	**Substrate**	**OER**		**HER**		**Overall water splitting cell voltage (10 mA cm^{-2})**	**References**
		**Overpotential (mV) at 10 mA cm^{-2}**	**Tafel slope (mV dec^{-1})**	**Overpotential (mV) at 10 mA cm^{-2}**	**Tafel slope (mV dec^{-1})**		
**Co_7Fe_3/Co-600**	**Ni foam**	**200 (90% iR)**	**59.4**	**68 (90 % iR)**	**55.8**	**1.50**	***This work***
Ni_3Fe/C	Carbon paper	201 (after iR)	82	70 (after iR)	78	1.54	*Adv. Funct. Mater.*, **2022**, *32*, 2109709.
$Co_{0.42}Fe_{0.58}P$@C	—	262 (90% iR)	44.8	181 (90 % iR)	66.6	1.55	*Adv. Energy Mater.* **2022**, *12*, 2202394.
$Fe_{0.4}Co_{0.3}Ni_{0.3}$-1.8	alloy plate	184 (95% iR)	48	175 (95 % iR)	46	1.62	*Energy Environ. Mater.* **2023**, *0*, e12590.
Ni_3Fe-NC/NF	Ni foam	203 (95% iR)	57.2	98 (95 % iR)	77.4	1.49	*J. Mater. Chem. A*, **2023**, *11*, 6452.

CoFe-250	Ni foam	230 (80% iR)	50	132 (80 % iR)	48	1.47	*Chem. Eng. J.*, **2022**, *432*, 134275.
Fe–Co–P/NF	Ni foam	227 at 20 mA cm^{-2} (100% iR)	55	87 (100 % iR)	63	1.55	*J. Mater. Chem. A*, **2021**, *9*, 24677.
CoFe-NA_2 /NF	Ni foam	259 (without iR)	69.9	73 (without iR)	96.7	1.564	*J. Energy. Chem.*, **2022**, *65*, 405.
CoFe PBA@CoP/NF	Ni foam	171 (after iR)	75.7	100 (after iR)	60.8	1.542	*Small Methods*, **2021**, *5*, 2100125.
FeCo-FeCoP@C@NCCs	Carbon cloth	280 (after iR)	58	91 (after iR)	38.3	1.64	*J. Energy. Chem.*, **2021**, *53*, 1.
CuNi@NiFeCu	Carbon paper	285 at 20 mA cm^{-2} (90% iR)	59	42 (90 % iR)	133	1.51	*Appl. Catal. B*, **2021**, *298*, 120600.
CuFe/NF	Ni foam	218 (after iR)	62.07	158 (after iR)	62.18	1.64	*Small*, **2020**, *16*, 1905884.
Ni_3FeN/Ni_3Fe	—	250 (95% iR)	51	125 (95 % iR)	98	1.61	*J. Mater. Chem. A*, **2021**, *9*, 4036.
CuNi@NiFeCu	Carbon paper	285 at 20 mA cm^{-2} (90% iR)	59	42 (90 % iR)	133	1.64	*Appl. Catal. B*, **2021**, *298*, 120600.
CoFe@N-CNTs-800	—	306 (after iR)	62	115 (after iR)	130	1.64	*J. Mater. Chem. A*, **2021**, *9*, 2174.

Table S2. Bonding energy and corresponding peak area percentage (ar.%) in Co 2p XPS spectra.

**Catalysts**	**Co^0 (eV)**		**ar.%**	**Co^{3+} (eV)**		**ar.%**	**Co^{2+} (eV)**		**ar.%**
	**$2p_{3/2}$**	**$2p_{1/2}$**		**$2p_{3/2}$**	**$2p_{1/2}$**		**$2p_{3/2}$**	**$2p_{1/2}$**	
Co_7Fe_3/Co-600	777.7	792.7	18.9	780.1	795.6	27.9	782.5	797.6	28.7
Post-OER	—	—	—	779.9	795.1	40.3	782.0	797.0	27.7
Post-HER	778.1	793.4	5.4	780.4	796.2	32.8	783.1	798.2	37.0

Table S3. Bonding energy and corresponding peak area percentage (ar.%) in Fe 2p XPS spectra.

**Catalysts**	**Fe^0 (eV)**		**ar.%**	**Fe^{2+} (eV)**		**ar.%**	**Fe^{3+} (eV)**		**ar.%**
	**$2p_{3/2}$**	**$2p_{1/2}$**		**$2p_{3/2}$**	**$2p_{1/2}$**		**$2p_{3/2}$**	**$2p_{1/2}$**	
Co_7Fe_3/Co-600	707.1	720.0	7.0	710.8	722.1	37.3	713.6	725.7	32.4
Post-OER	—	—	—	711.1	722.7	29.6	714.2	726.3	41.4
Post-HER	706.8	719.7	8.5	710.6	721.6	32.4	713.2	725.3	33.7

Table S4. Bonding energy and corresponding peak area percentage (ar.%) in O 1s XPS spectra.

Catalysts	M-O		-OH		O=C/C-O-C		Ads.H_2O	
	position	ar.%	position	ar.%	position	ar.%	position	ar.%
Co_7Fe_3/Co-600	529.8	20.9	531.4	42.3	532.9	36.8	—	—
Post-OER	529.6	8.9	531.2	65.4	532.8	17.5	535.2	8.2
Post-HER	530.0	11.7	531.6	40.4	533.1	27.2	535.3	20.7

Table S5. The Gibbs free energy of O-intermediates and hydrogen at different sites.

Absorbed sites	OER				HER
	$\Delta G_1=\Delta G_{*OH}$ (eV)	$\Delta G_2=\Delta G_{*O}-\Delta G_{*OH}$ (eV)	$\Delta G_3=\Delta G_{*OOH}-\Delta G_{*O}$ (eV) RDS	$\Delta G_4=\Delta GO_2-\Delta G_{*OOH}$ (eV)	$\lvert\triangle G_{H*}\rvert$ (eV)
Co_7Fe_3/Co Co site in Co_7Fe_3	0.730	0.917	1.662	1.610	0.246
Co_7Fe_3/Co Fe site in Co_7Fe_3	0.749	1.113	1.732	1.326	0.201
Co_7Fe_3/Co Co site in metallic Co	0.463	0.897	2.109	1.451	0.458
Co_7Fe_3/Co site at interface	0.650	1.427	1.460	1.383	0.146
pure-phase Co_7Fe_3 Co site	1.138	1.412	2.003	0.367	0.479
pure-phase Co_7Fe_3 Fe site	0.754	1.214	2.033	0.918	0.520

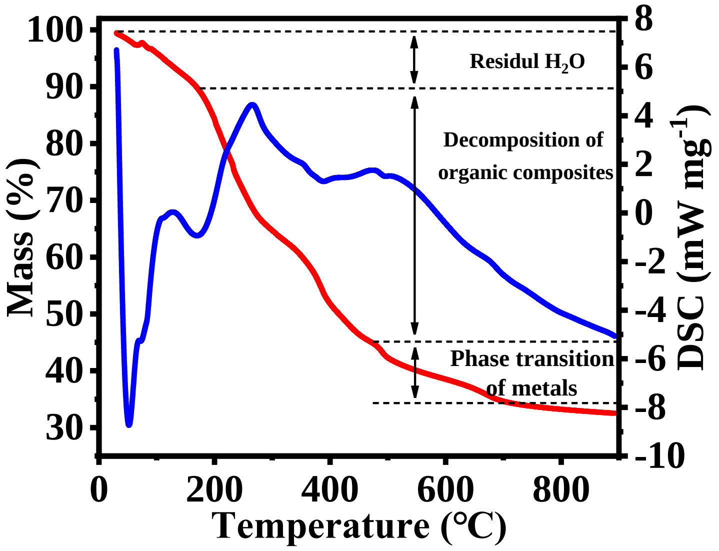

Figure S1. TG-DSC curves for the decomposition of PVP-metal ion precursor at N_2 atmosphere.

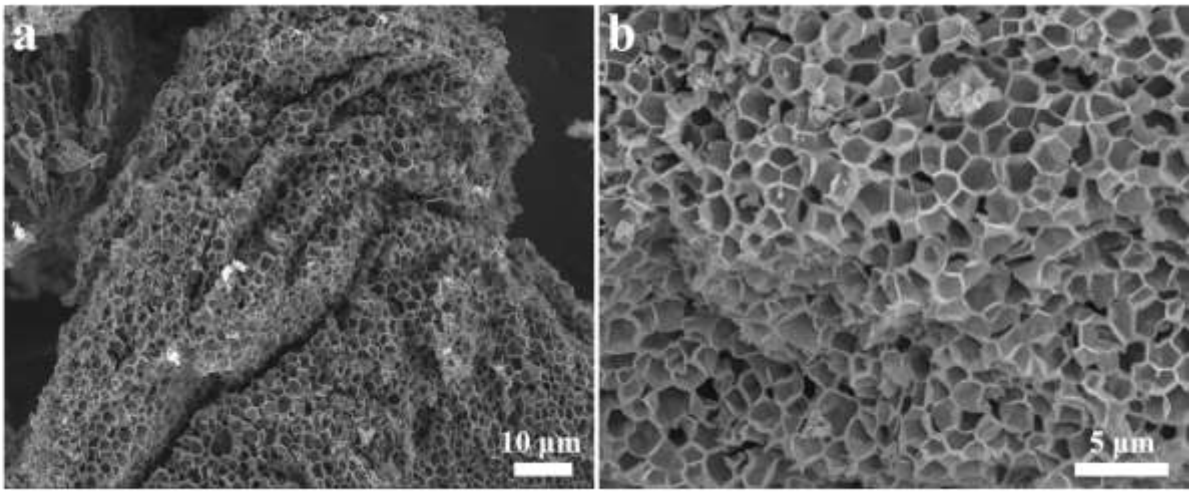

Figure S2. The low-magnification SEM images of honeycomb structure.

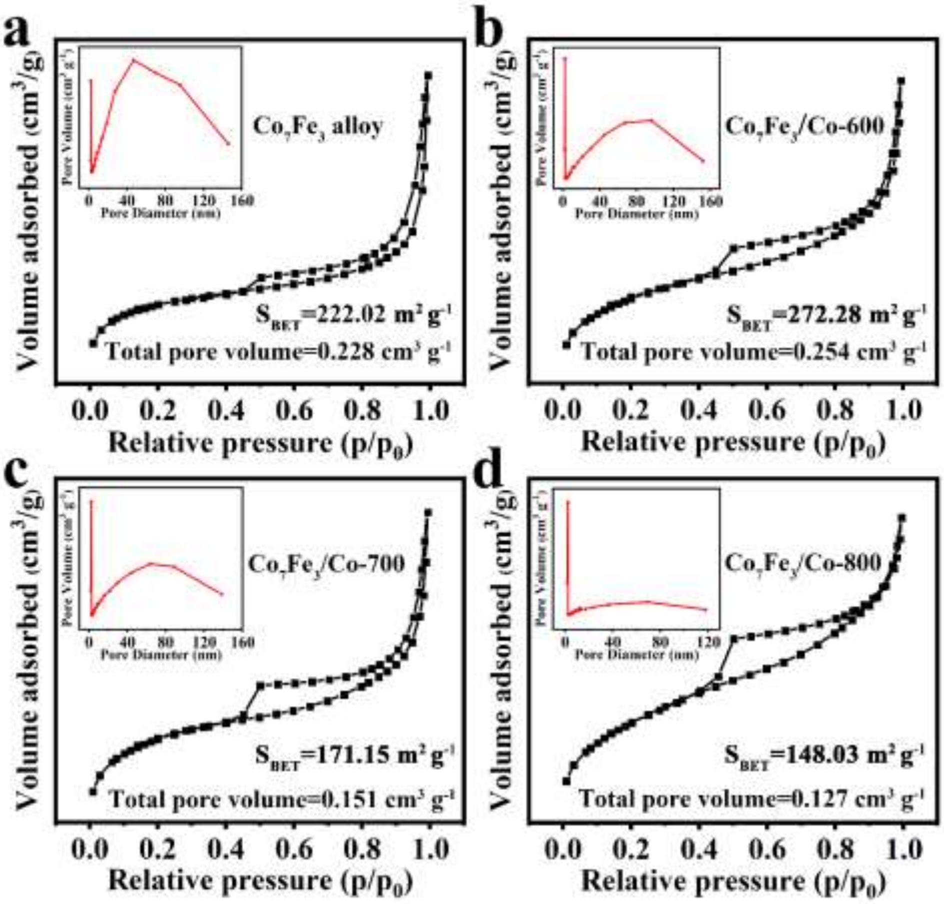

Figure S3. N_2 adsorption-desorption isotherms and pore size distributions (inset) for (a) pure-phase Co_7Fe_3 alloy, (b) Co_7Fe_3/Co-600, (c) Co_7Fe_3/Co-700 and (d) Co_7Fe_3/Co-800.

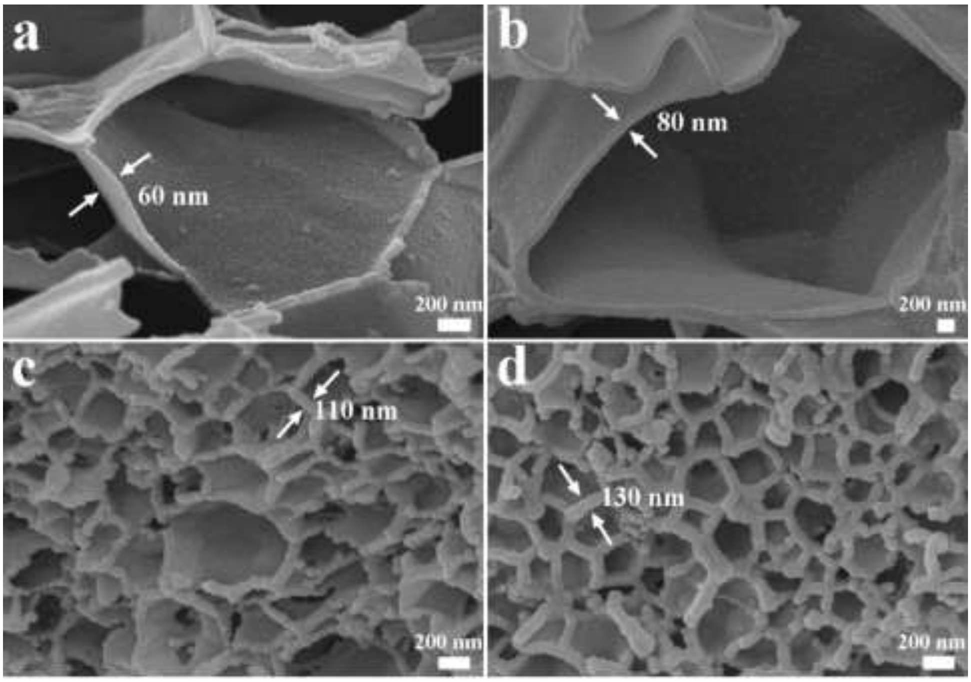

Figure S4. The SEM images of (a) pure-phase Co_7Fe_3 alloy, (b) Co_7Fe_3/Co-600, (c) Co_7Fe_3/Co-700 and (d) Co_7Fe_3/Co-800.

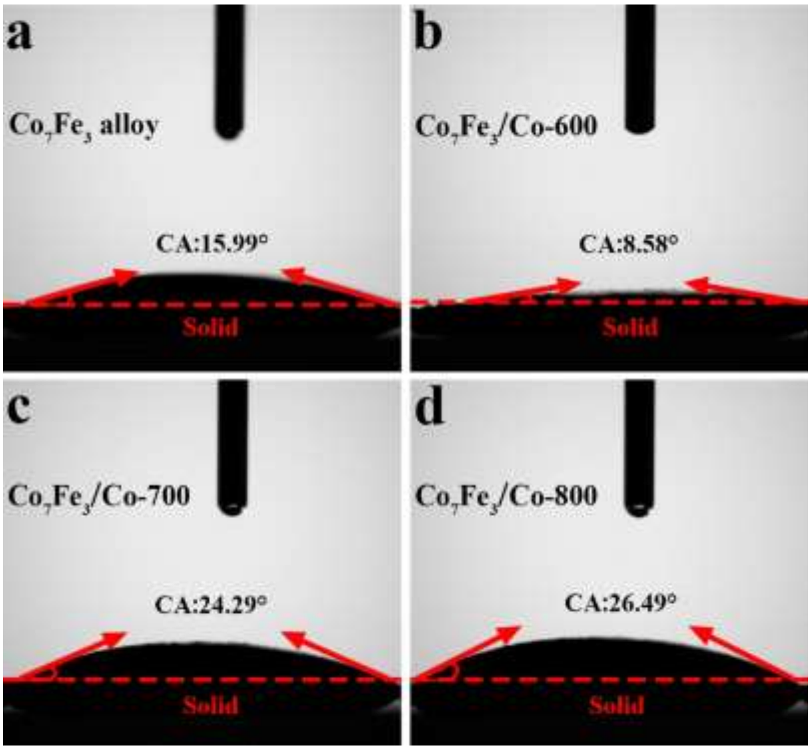

Figure S5. The contact angle measurements for (a) pure-phase Co_7Fe_3 alloy, (b) Co_7Fe_3/Co-600, (c) Co_7Fe_3/Co-700 and (d) Co_7Fe_3/Co-800.

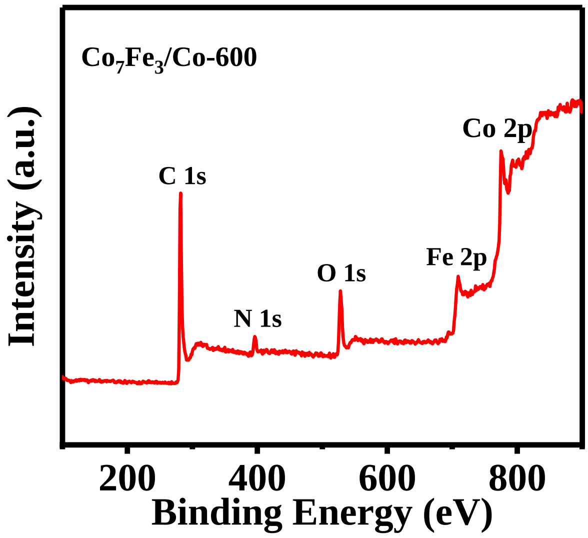

Figure S6. Full-XPS spectrum of Co_7Fe_3/Co-600 sample.

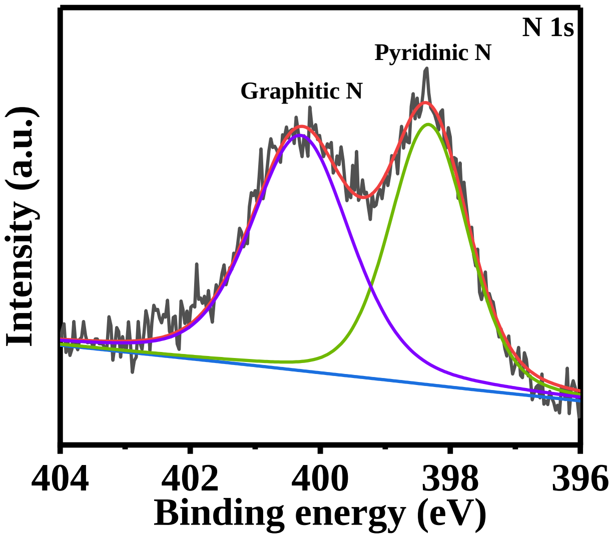

Figure S7. High resolution XPS spectrum of N 1s for Co_7Fe_3/Co-600 sample.

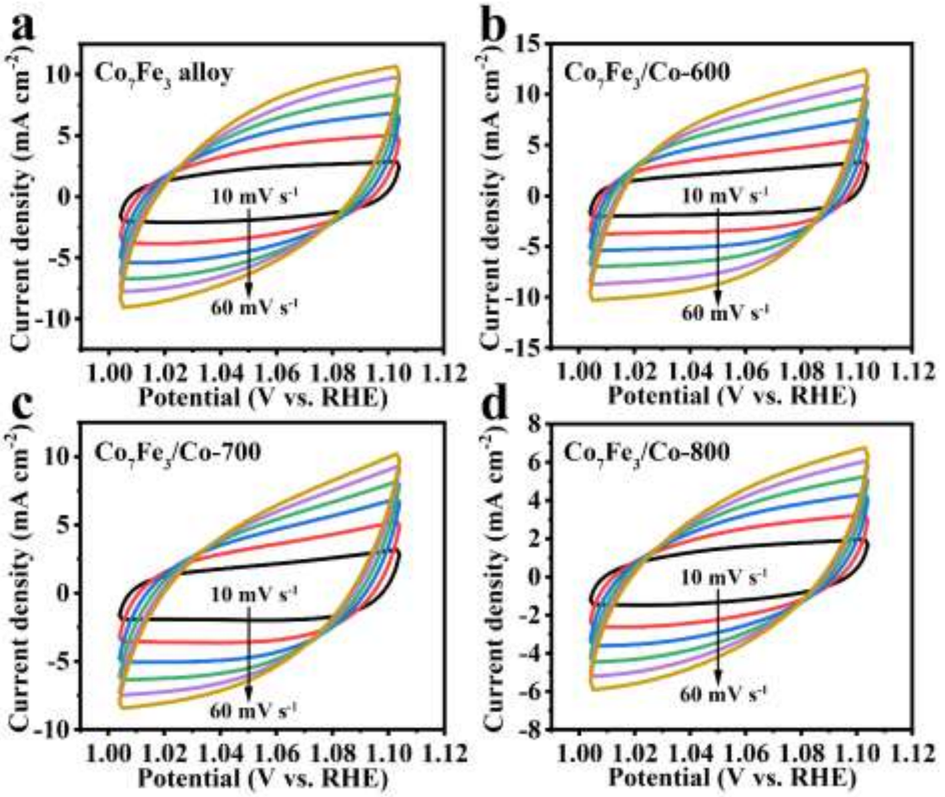

Figure S8. Cyclic voltammograms (CVs) of (a) pure-phase Co_7Fe_3 alloy, (b) Co_7Fe_3/Co-600, (c) Co_7Fe_3/Co-700 and (d) Co_7Fe_3/Co-800 for OER with sweep rates in the range of 10-60 mV s^{-1} in 1.0 M KOH.

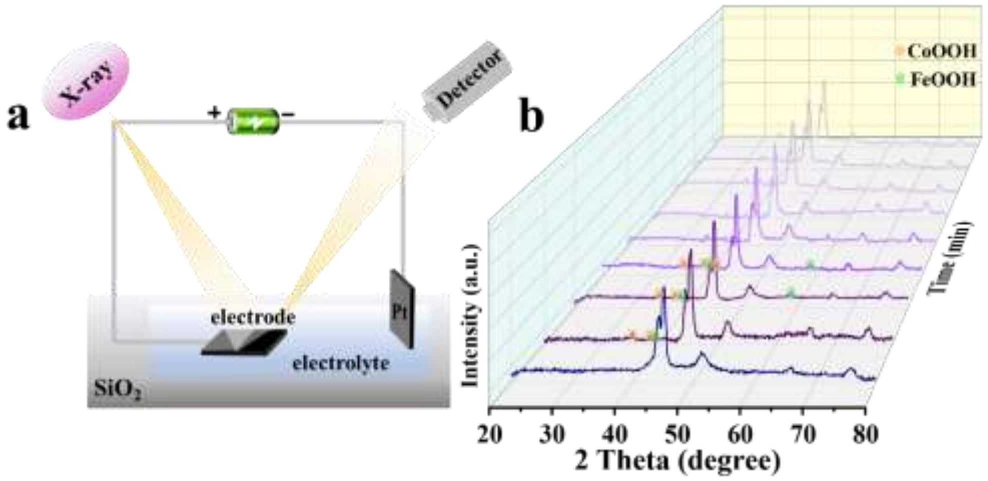

Figure S9. (a) Schematic diagram of the XRD diffractometer equipped with a two-electrode electrolysis system for in situ XRD measurement; (b) In situ XRD patterns of Co_7Fe_3/Co-600 collected during OER.

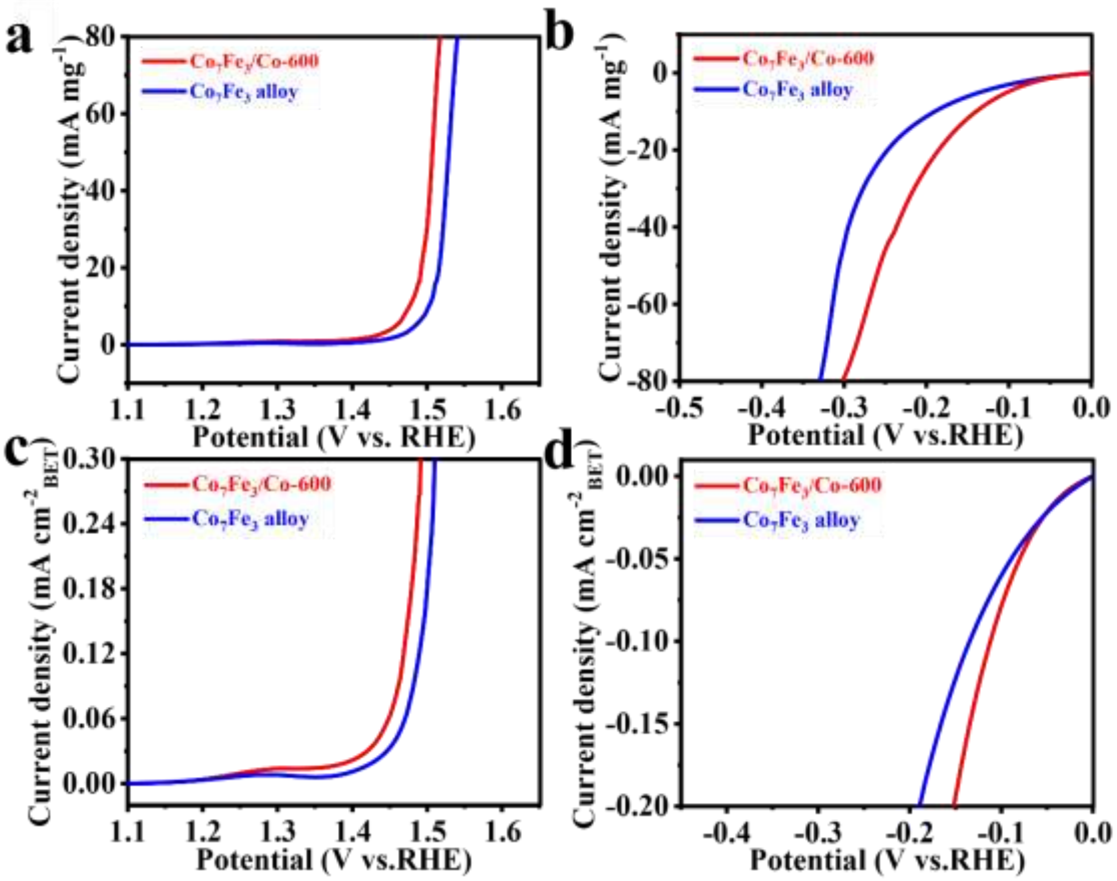

Figure S10. Polarization curves of Co_7Fe_3/Co-600 and pure-phase Co_7Fe_3 alloy for OER and HER normalized by loading weight of catalyst on electrode and surface areas, respectively.

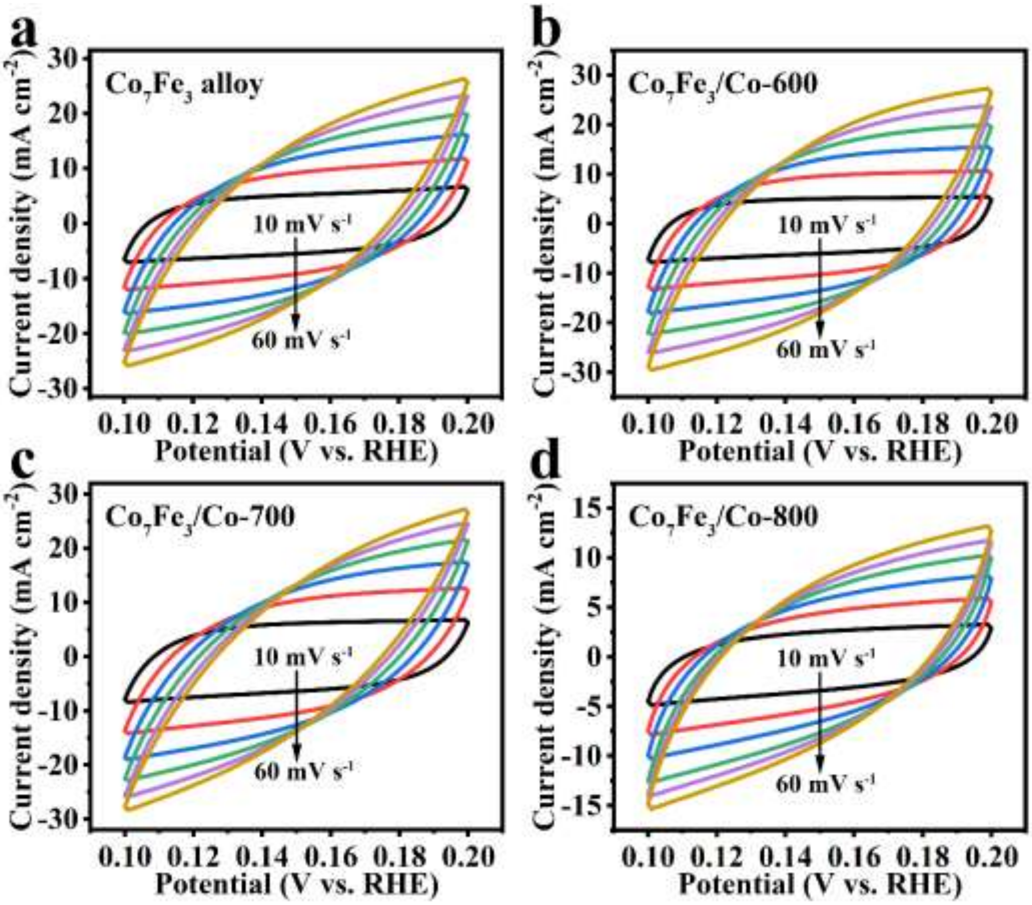

Figure S11. Cyclic voltammograms (CVs) of (a) pure-phase Co_7Fe_3 alloy, (b) Co_7Fe_3/Co-600, (c) Co_7Fe_3/Co-700 and (d) Co_7Fe_3/Co-800 for HER with sweep rates in the range of 10-60 mV s^{-1} in 1.0 M KOH.

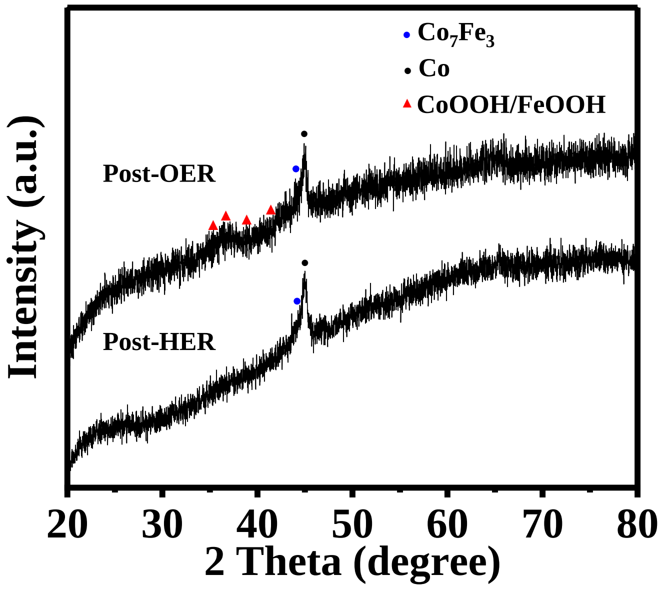

Figure S12. XRD patterns of Co_7Fe_3/Co-600 after OER and HER stability test.

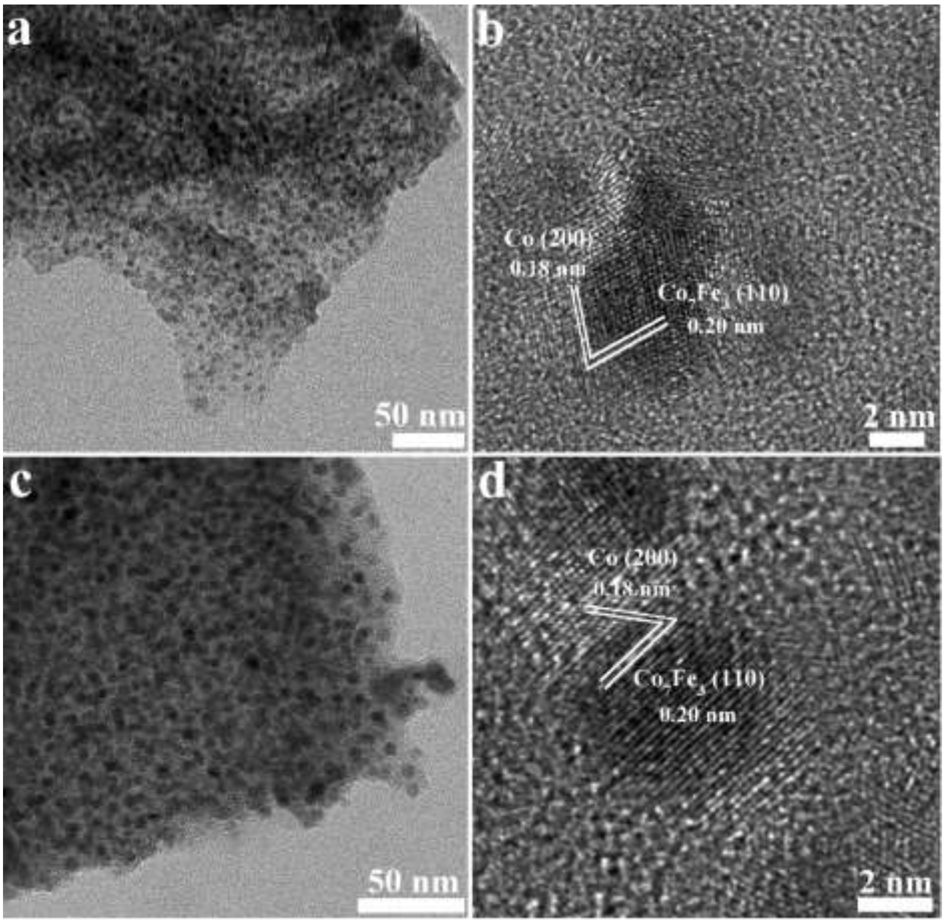

Figure S13. TEM and HRTEM images of Co_7Fe_3/Co-600 after (a, b) OER and (c, d) HER stability tests.

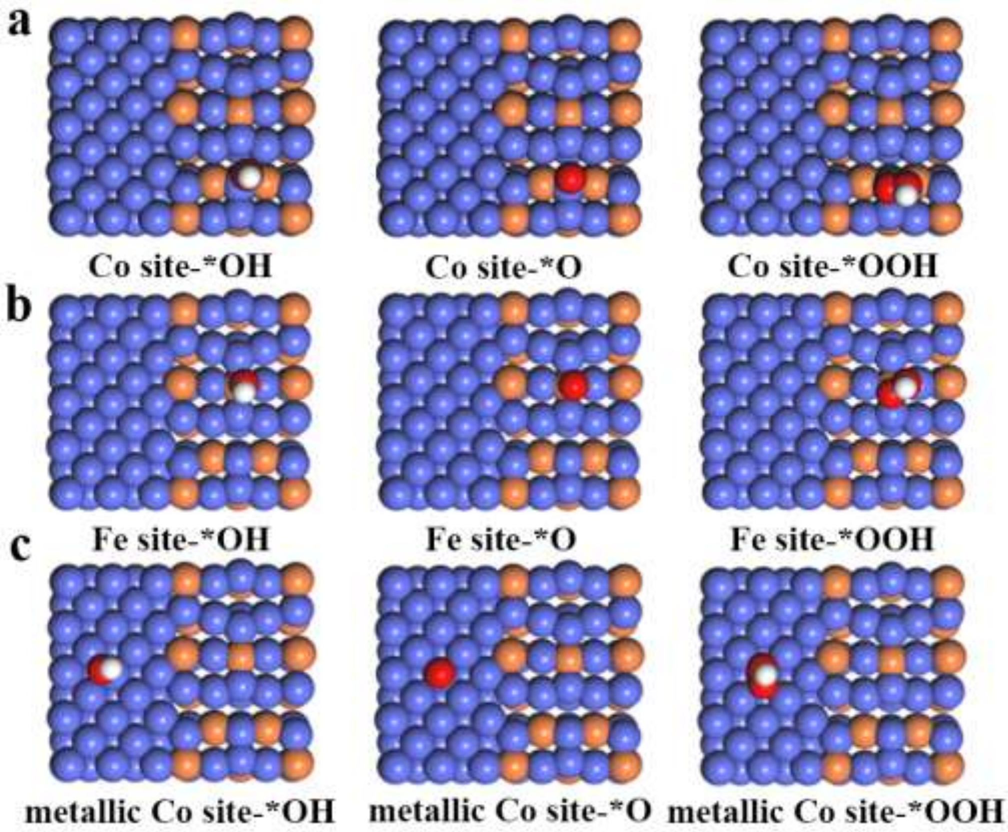

Figure S14. The optimized structures of *OH, *O and *OOH intermediates adsorbed on (a) Co site, (b) Fe site and (c) metallic Co site in Co_7Fe_3/Co sample.

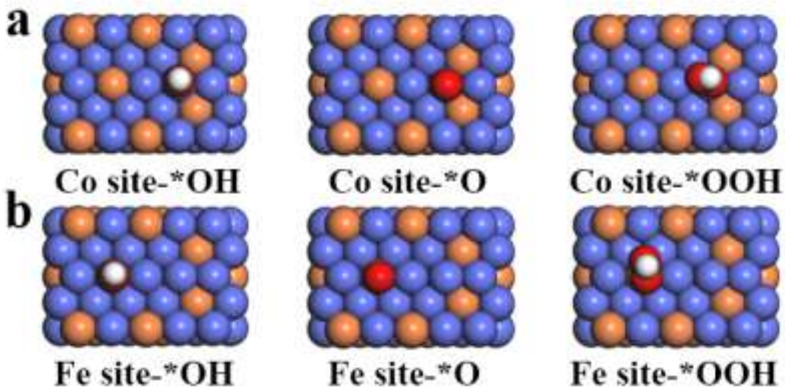

Figure S15. The optimized structures of *OH, *O and *OOH intermediates adsorbed on (a) Co site and (b) Fe site in pure-phase Co_7Fe_3 alloy.

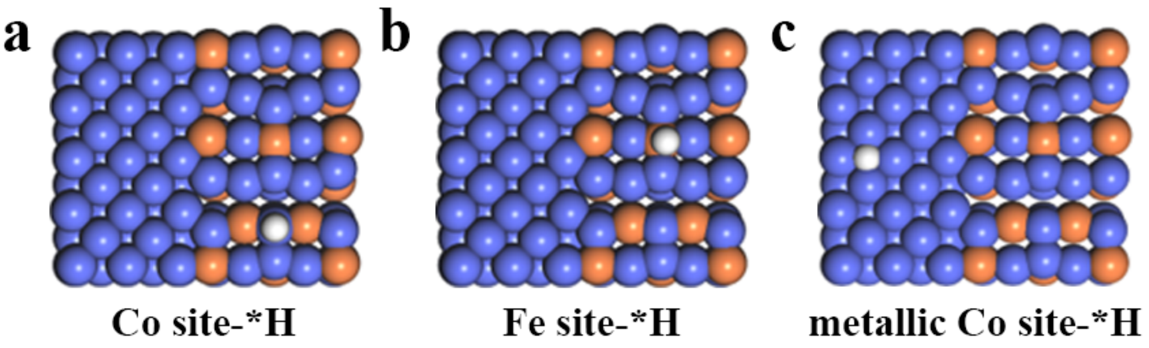

Figure S16. The optimized structures of *H intermediate adsorbed on (a) Co site, (b) Fe site and (c) metallic Co site in Co_7Fe_3/Co sample.

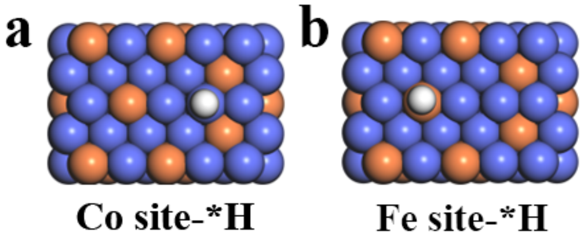

Figure S17. The optimized structures of *H intermediate adsorbed on (a) Co site and (b) Fe site in pure-phase Co_7Fe_3 alloy.

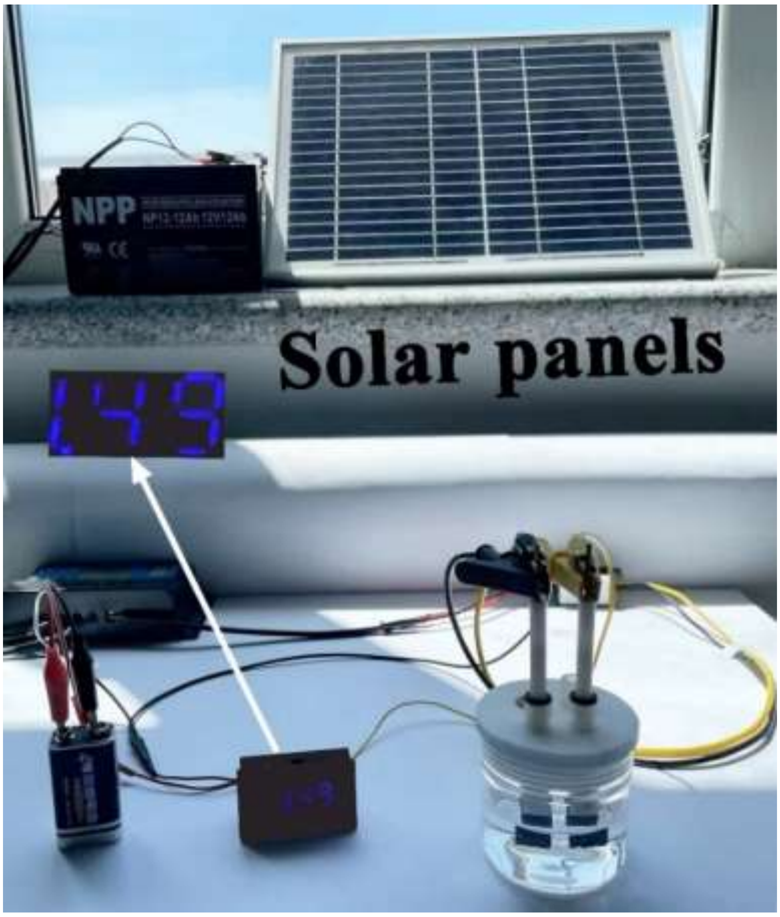

Figure S18. The water splitting device driven by a solar cell with a voltage of 1.49 V.

References

[1] S. Anantharaj, S. R. Ede, K. Karthick, S. Sam Sankar, K. Sangeetha, P. E. Karthikc, S. Kundu, *Energy Environ. Sci.*, **2018**, *11*, 744.

[2] C. C. McCrory, S. Jung, J. C. Peters, T. F. Jaramillo, *J. Am. Chem. Soc.* **2013**, *135*, 16977.

[3] Y. Liu, Y. Chen, Y. Tian, T. Sakthivel, H. Liu, S. Guo, H. Zeng, Z. Dai, *Adv. Mater.* **2022**, *34*, 2203615.

[4] G. Kresse, D. Joubert, *Phys. Rev. B* **1999**, *59*, 1758.

[5] J. P. Perdew, K. Burke, M. Ernzerhof, *Phys.Rev. Lett.* **1996**, *77*, 3865.

[6] S. Grimme, J. Antony, S. Ehrlich, H. Krieg, *J. Chem. Phys.* **2010**, *132*, 154104.
